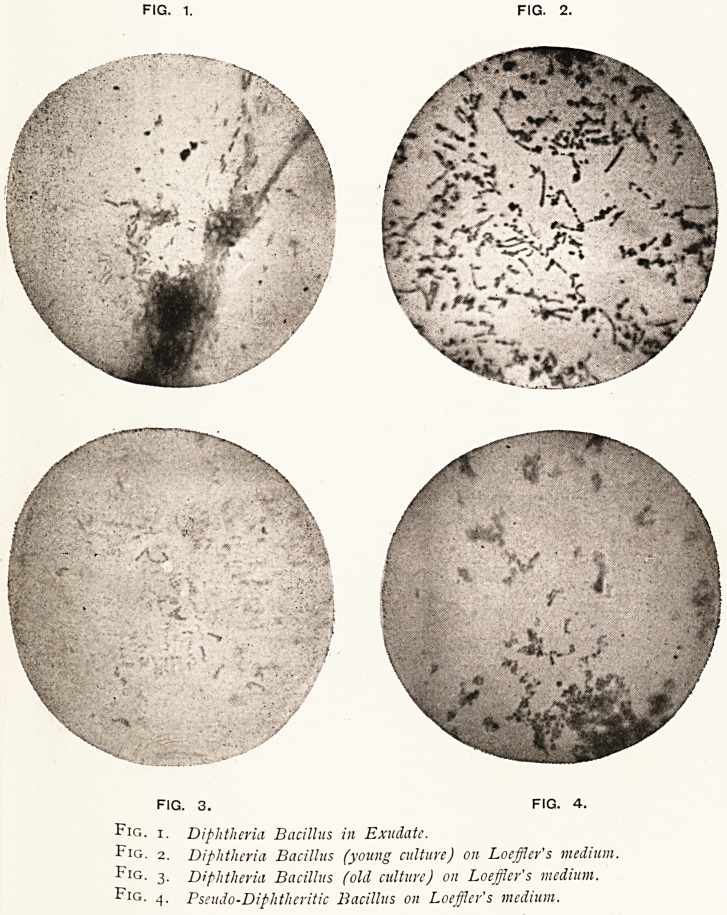# The Bacterioscopic Diagnosis of Diphtheria

**Published:** 1895-03

**Authors:** F. Wallis Stoddart

**Affiliations:** Lecturer in Hygienic Chemistry and Bacteriology in University College, Bristol; Public Analyst for the City of Bristol


### THE BACTERIOSCOPIC DIAGNOSIS OF DIPHTHERIA.

BY

F. Wallis Stoddart, F.I.C., F.C.S.,

*Lecturer in Hygienic Chemistry and Bacteriology in University College, Bristol; Public Analyst for the City of Bristol.*

The positive detection of any specific micro-organism depends: first, upon the possession of thoroughly trustworthy methods of separation from other schizomycetes; and secondly, on a knowledge of the characteristic features, both cultural and morphological, by means of which the microbe under observation may be satisfactorily identified when isolated.

In the case of the Klebs-Loeffler bacillus, the mode of separation and the behaviour under cultivation are so intimately connected, that it will be convenient for the present purpose to consider them together after reviewing the morphological features.

*Morphological Character of the Diphtheria Bacillus.*—When observed in cover-glass preparations of exudate, this organism appears as a typical bacillus, the length considerably exceeding the breadth, which under the usual conditions of staining is about 0.7 $\mu$. The rods are straight or slightly bent, do not grow into threads, but are frequently aggregated in knots or skeins embedded in mucous bands, which take a light stain. (Fig. 1.) It is quite rare in the early stages of the disease, during which the material is or should be submitted to examination, to find the bacilli otherwise than uniform in diameter and evenly stained; and any attempt to recognise the special appearance described as characteristic of the bacillus will probably result in disappointment.

In later stages this uniformity of appearance is lost in a greater or less degree, and rods may be found approximating in appearance to those grown on artificial media; and in *post-mortem* sections, when the death of the patient has ensued at some considerable time subsequent to the attack, this similarity may be very complete.

So commonplace is the aspect of the bacilli in the exudate, that it is only when they are unusually abundant that it is possible to distinguish them with any degree of certainty from very similar organisms which occur in non-specific affections of the throat; and then it is rather the mode of aggregation than the microscopic appearance that guides the observer.

It will generally be observed that numerous other organisms, especially forms of coccus, accompany the diphtheria bacillus; but the former are almost always thronging around epithelial cells and similar *débris*, and are not so distinctly embedded in the mucous threads.

The bacilli are readily stained by the usual simple stains, but are somewhat more readily decolorised by washing than is the case with other organisms. Gram's method also gives excellent results, especially in sections.

The above description applies almost precisely to the bacillus grown in peptone broth at blood temperature; but here there is not infrequently some irregularity of form, chiefly in the shape of enlarged extremities. It is, however, when grown on solid media, and especially on blood-serum, that it develops its most striking morphological characteristics. Rods from young growths (twenty-four hours) present very densely-stained spherical bodies, one of which is generally situated at either extremity, whilst others are placed at intervals in the length of the bacillus: the diameter is apparently increased at such spots, especially at the ends, the intervening portions being narrower and comparatively faintly stained; a very characteristic appearance is that of a faintly-stained prolongation of the bacillus terminated by one of the deeply-stained granules. (Fig. 2.) These appearances are best brought out by staining with methylene blue dissolved in weak alkali (.01 per cent. caustic potash), or in five per cent. carbolic acid, or by Gram's method; but the enlarged extremities are particularly conspicuous in fuchsine-stained preparations. In older cultures the differentiation becomes more and more marked, until it requires close observation to detect the intermediate portions of the bacillus, the granules appearing as blue-black points connected by faint bluish bonds. The whole appearance is now suggestive of a streptococcus, the chief distinction being the interval between adjacent granules. (Fig. 3.) Well-marked varieties as regards length are found in cultures from different cases, and are apparently constant; but I have not found the same difference in the exudates from which the cultures were made.

*Cultural Characters of the Diphtheria Bacillus.*—This organism requires for its active growth a temperature of 30° C. and upwards, flourishing best at about 37° C. It grows luxuriantly in the ordinary alkaline peptone broth, the reaction of which it modifies in a most peculiar way. The broth becomes progressively acid up to about forty-eight hours, after which the action is reversed, until at the end of a week the culture is strongly alkaline. This change is well shown by the addition of an indicator to the broth: for this purpose, litmus purified by precipitation with alcohol is excellently adapted.

Of the solid media, gelatine is the least satisfactory, owing

FIG. 1.

FIG. 2.

FIG. 3.

FIG. 4.

FIG. 1. *Diphtheria Bacillus in Exudate.*
FIG. 2. *Diphtheria Bacillus (young culture) on Loeffler's medium.*
FIG. 3. *Diphtheria Bacillus (old culture) on Loeffler's medium.*
FIG. 4. *Pseudo-Diphtheritic Bacillus on Loeffler's medium.*

to its low melting-point; on agar and glycerine agar also the growth is very slow, though characteristic in appearance. Blood-serum, however, especially as modified by Loeffler—that is, with the addition of one-third glucose broth,—is excellent, the growth not only being copious in less than twenty-four hours, but anticipating that of many foreign organisms, whilst the protoplasmic differentiation is strongly accentuated. It should be noted that a very practical improvement in the preparation of serum media was effected by the recognition of the fact that there is little or no advantage to be gained by securing transparency: the method of slow sterilisation formerly adopted is therefore superseded by the usual form of steam sterilisation, whereby much time and trouble are saved.

On the Loeffler medium, kept at 37° C., colonies make their appearance within twenty-four hours after inoculation as small, moist-looking elevations, more or less discrete according to the amount of material introduced. They slowly increase in size for a few days, but never attain a greater diameter than about 2 mm., and frequently remain much smaller; as they become older they dry somewhat, the centre becoming acuminate, and radiating ridges make their appearance. In favourable cases no other colonies result from inoculation with exudate, even when the latter contains many species of organisms; and it has been stated that this particular medium is so suited to the requirements of the diphtheria bacillus, that any colonies making their appearance within twelve to twenty hours must consist of this organism, and no other. This is hardly correct; for not infrequently colonies of cocci, and occasionally of yeasts, grow concurrently with the diphtheria bacillus, and so far resemble the latter that microscopic observation is necessary to discriminate between them. To this end, it is very desirable that the amount of material used for inoculation be not so large as to crowd the colonies, a result which generally follows the direct use of the swab. Hence it appears desirable to inoculate with a platinum loop rubbed over the swab, and then used for two or three tubes in succession. Both methods of procedure have been followed in each case examined by me, and in every case the bacillus was as readily detected when the inoculation was effected by the needle as by the usual method, whilst the ease with which particular colonies could be selected was of course far greater.

At the same time, it is true that most of the mouth bacteria, especially those which morphologically resemble the diphtheria bacillus, are eliminated by this method of culture. The change of reaction may be followed, as in the case of broth, by staining the medium with litmus; and a most interesting appearance is presented at the stage at which the red colonies are contrasted with the as yet unaltered blue background of medium. Sub-cultures may now be made on the other media in confirmation; but in the very large majority of cases the procedure so far described is quite sufficient to determine the presence of the diphtheria bacillus, as no other known organism conforms in every respect to the description given.

It is necessary, however, to refer briefly to the so-called pseudo-diphtheritic bacillus.

*The Pseudo-Diphtheritic Bacillus.*—At least two distinct organisms have been included under this name. One is in every respect similar to that already described, except that it is not virulent for animals; the other, an organism presenting such points of resemblance as to necessitate a careful examination, but at the same time capable of being sharply distinguished.

The former is almost certainly merely an attenuated form of the diphtheria bacillus, such as is known to be produced under circumstances not entirely favourable to its growth, and does not need further description.

The latter, however, is an interesting organism occasionally found in morbid throats. It has occurred only once in my experience, and presents the following distinctive features: The growth on Loeffler's medium is not unlike that of the true bacillus, but rather more abundant and confluent; on agar and gelatine it grows luxuriantly at ordinary temperatures; in broth it also grows well, but acidifies less rapidly and intensely, and does not present the subsequent reverse action; microscopically it exhibits differentiation of the protoplasm somewhat similar to that of the true bacillus, but although in each field there are individuals of which it could not be said from their appearance that they were not Klebs-Loeffler bacilli, very many taper uniformly from end to end, resembling in outline a Florence flask rather than a knotted stick. These almost always occur in pairs side by side, of which one individual is larger than the other. (Fig. 4.)

Thus the resemblance between the true and false bacilli is rather superficial, and should not confuse a careful observer.

It may be worth while to note that the rarity of its occurrence shows pretty clearly that the pseudo-diphtheritic bacillus bears no causal relation to the condition known as pseudo-diphtheria, and it would be conducive to a clearer understanding if both names were allowed to lapse.

*Practical Results.*—I have examined twenty-one cases, none of which were treated with serum, and the results were briefly as follows:

| | |
|---|---|
| Diphtheria bacillus found in ... ... ... ... | 12 |
| Long variety occurred in ... ... ... | 7 |
| Short ,, ,, ... ... ... | 5 |
| Fatal cases—Long variety ... ... ... | 5 |
| ,, Short ,, ... ... ... | 1 |

The proportion of cases in which the bacillus was found is somewhat lower than has been recorded by others; but this is in part due to the inclusion of some which would probably not have been definitely diagnosed as diphtheria on clinical grounds. With reference to the influence of the variety of bacillus present, I have to add to the figures given above that one of the two cases exhibiting the long variety in which recovery took place was exceedingly severe, and required the performance of tracheotomy: these cases, therefore, so far at least as is possible with so limited a number, support the distinction drawn by Dr. Washbourn and others between the long and short varieties as regards virulence.

I am unable to throw any light upon the question of mixed infection: it appears, however, that a more minute investigation requires to be made into the nature of the other organisms present in the exudate, but not appearing on the culture tubes.

Several of the above cases were examined more than once, and the description and proportion of accompanying organisms varied exceedingly at different dates.

A considerable number of normal and other throats have been examined in addition to the above, but in no case was any organism discovered at all resembling the diphtheria bacillus, except in one instance when the pseudo-diphtheritic bacillus above described was found.

I conclude, therefore, that bacterioscopic examination constitutes a practical and rapid method of discrimination between cases of throat affection which are, and those which are not, due to the attack of the diphtheria bacillus; and that it affords information of so much value, quite apart from indications as to treatment, as to justify its systematic application.

## Figures and Tables

**Fig. 1. Fig. 2. Fig. 3. Fig. 4. f1:**